# Relation of income to trends in well-being by age: implications for the future older “forgotten” lower middle class

**DOI:** 10.1093/haschl/qxae183

**Published:** 2025-01-09

**Authors:** David H Rehkopf, Frank F Furstenberg, Christian Jackson, John W Rowe, and The Research Network on an Aging Society, John W Rowe, Toni C Antonucci, Lisa Berkman, Axel Börsch-Supan, Laura L Carstensen, Cynthia Chen, Dana P Goldman, Linda P Fried, Frank F Furstenberg, Martin Kohli, S Jay Olshansky, David H Rehkopf, Julie Zissimopoulos

**Affiliations:** Department of Epidemiology and Population Health, Stanford University, Palo Alto, CA 94304, United States; Department of Sociology, University of Pennsylvania, Philadelphia, PA 19104, United States; Department of Epidemiology and Population Health, Stanford University, Palo Alto, CA 94304, United States; Deparement of Health Policy and Management,Columbia University, New York, NY 10032, United States

**Keywords:** well-being, income, aging, retirement

## Abstract

The structure of social welfare policy has neglected a growing and increasingly economically marginalized segment of the American population—the lower middle class, a large group who are ineligible for many need-based social services. We examined 20-year time trends in physical well-being, mental well-being, and functional well-being by levels of household income. Our descriptive study used data from the Behavioral Risk Factor Surveillance System and is representative of the population of the United States, ages 40 to 74, from 2003 to 2022 (*n* = 5 308 256). We found dramatic and consistent differences in trends in well-being by income category. While well-being generally got worse over the 20-year period for all ages, the declines were most pronounced for lower-middle-income households for individuals age 50 to 59. These differential trends by income were similar for all 3 of the measures of well-being we examined, but were most different by income level for physical well-being and functional well-being. No major trends or levels were explained by race, body mass index, or smoking. If the observed trends persist, the current age 50–59-year-old lower-middle-income population will enter retirement ages with substantially worse well-being than previous generations.

## Introduction

The United States’ social welfare system is unique among industrialized countries and includes both labor market– and non–labor market–dependent supports. With respect to the labor market, since the “welfare to work” reforms of the 1990s, the largest and most effective poverty reduction policy for working families in the United States has been the Earned Income Tax Credit, which distributes approximately 70 billion dollars per year to families—but only to households with earnings.^1^ The remaining focus of social welfare programs is primarily on individuals in households at lower levels of income—most typically those households living below 185% of the federal poverty line.^2^ Medicaid is one such notable program where primarily only low-income households qualify.^3^ This structure of social welfare policy has neglected a growing and increasingly economically marginalized part of the American population—the lower middle class. This is particularly important from a health and aging perspective, as prior work has shown that health is not just associated with poverty but that the strong association between household income and health exists up to near-median income.^4-6^ Recent work has shown that there are particularly low levels of health and well-being prior to retirement among those households between the 15th and the 45th percentiles of household income, a group referred to as the “forgotten middle.”^7^ This finding is particularly troubling given the overall societal and economic implications of such a large proportion of the US population entering retirement without the health or financial resources to be engaged and productive.

A healthy and productive aging society benefits from the concept of “compression of morbidity.”^8,9^ This is the proposed scenario where, as population life expectancy increases, there are also fewer years of disability and functional limitations in the population. This theory has been the operating assumption behind many of our social policies, that the population would grow healthier with less medical supports and more labor force participation as life expectancy increased. If, however, morbidity actually increases more rapidly than life expectancy, morbidity decompresses, with important implications for many social policies that impact those leaving the labor force, with particular impacts on Medicare expenditures.

Several important questions about these recent findings on increasing morbidity for the lower-middle class, however, need to be clarified to understand the current situation and potential future trends. Prior work focused on pre-retirement ages 53 to 58, given the importance of health and resources in the years immediately prior to retirement. However, it is well recognized from life-course aging theory that exposures up to several decades before retirement impact health and well-being after retirement.^10^ The trajectory of trends of the health of the lower middle class is also relatively unknown and requires the use of data with similar measures on a regular basis over decades to adequately describe these trends. In addition, it is unclear whether differences in current levels of health and well-being and trends across time by household level are primarily driven by differences in well-known other factors driving income differential in health, such as race, as an indicator of exposure to structural racism, obesity, and smoking. Prior work on trends in well-being at ages before and after retirement was not in datasets with large-enough sample sizes to look at year-specific trends by income level and race and ethnicity. Examining these differences is an important first step to understanding which populations are most at risk in these declines and can help give important insights into potential drivers of these trends that could be investigated in future work.

To further our understanding of the differences and trends in health and well-being by household income and age, with a focus on the lower-middle-income US population, we examined data on trends in health and well-being from the Centers for Disease Control and Prevention's (CDC’s) Behavioral Risk Factor Surveillance System (BRFSS). The BRFSS is a nationally representative yearly cross-section of the US population, taken from all 50 states, that has asked similar questions about health and well-being since 2003. The very large sample size, including 5.3 million people from 2003 to 2022, permits analyses of representative cross-sectional populations across time. We examined trends in self-reported physical well-being, mental well-being, and overall functional well-being by household income categories, from 2003 to 2022, including models that control for race and ethnicity, obesity, and smoking, and that stratify trends by race and ethnicity.

## Data and methods

### Data source

Data from years 2003 to 2022 were obtained directly from CDC's BRFSS (www.cdc.gov/brfss). All data used in our analyses are publicly available.

### Study population

Our study population was the noninstitutionalized population of the United States, ages 40 to 74, from 2003 to 2022. The age range was chosen to represent a substantial range of ages prior to typical retirement age, to better understand the health and well-being of the population entering retirement over the next 2 decades. We performed sensitivity analysis estimating trends from 2003 to 2019 to examine whether trends were impacted by COVID-19–related changes in 2020 through 2022. To best facilitate comparison with other work, we used the broad combined race and ethnicity categories from BRFSS: American Indian and Alaska Native (AIAN), Asian, Black, Hispanic, Other, and White. We used race and ethnicity as social categories that capture differential exposures experienced by minoritized populations in the United States.^11^ The BRFSS sample size for ages 40 to 74, from 2003 to 2022, was 5 308 256. Among those people with known ages, there were 1 007 808 individuals (19.0% of the sample) missing data on at least 1 of the 4 covariates (race and ethnicity, body mass index [BMI], smoking, income). Table S1 presents information on missing data on income by year and Table S2 presents data on missing data counts by model and outcome. Missing data on income range from 12% to 21% per year.

### Exposure

We defined household income categories by yearly income percentiles to capture 4 salient parts of the income distribution as closely as is possible to be consistent with prior work^7^: 0%–15%, 16%–45%, 46%–75%, and 76%-100%, which we refer to as “low,” “lower-middle,” “upper-middle,” and “high.” To minimize age-related confounding of distributions by income, we calculated percentiles of household income specific to our primary age strata of 40–49, 50–59, and 60–74 years, and equivalized for household size by dividing income by the square root of household size.^12^ Income and age categories were selected to be consistent with the prior work by Chapel et al.^7^ Details of the use of BRFSS data to create these household income categories are presented in the Tables S3–S5. Briefly, we used midpoints of the income categories presented in Table S3, and estimated the midpoint of the upper open-ended income category using a Pareto distribution.^13^ After equivalizing for household size, the proportion of the sample in each of the income categories was very close to that of the optimal distribution (Table S4), and the differences in distribution did not vary markedly over time (Table S5). For example, in our population of focus, the lower-middle-income category, the proportion of the population in this income category ranged from 27% to 38% by year, and this variation did not have any temporal trends that would be associated with our observed findings.

### Outcome

Our choice of outcomes was based on using measures that (1) are validated population-based measures of health and well-being, (2) capture several different domains of health and well-being, (3) meaningfully capture well-being across a wide range of ages, and (4) are measured consistently in the population across several decades in a nationally representative US sample. Based on these criteria, we selected the following 3 measures, which are part of the CDC's Health Related Quality of Life (HRQOL) battery of measures, have been validated, and while correlated with each other, have been shown to capture unique aspects of well-being^14,15^:

1. Physical well-being: Number of days in past 30 days in which physical health is not good (range: 0 to 30)
2. Mental well-being: Number of days in past 30 days in which mental health is not good (range: 0 to 30)
3. Functional well-being: Number of days in past 30 days in which poor physical or mental health kept you from doing usual activities such as self-care, work, or recreation (range: 0 to 30)

### Statistical analysis

Our primary models were fit with interactions between age category, year, and income category (base model). Our secondary models controlled for race and ethnicity, BMI, smoking, smoking + BMI, and race and ethnicity + smoking + BMI. We fit parallel additional models, but with interactions between year, income, age, and race and ethnicity, to examine whether the impacts of income level differed by race and ethnicity. All analyses used BRFSS sampling weights. The BRFSS provided BMI as a precalculated continuous variable, which is derived using weight (kg) and height (m). We transformed this into a categorical variable with 5 levels: <18.5, 18.5–24.9, 25.0–29.9, 30.0–34.9, and 35.0+. Smoking status is a categorical variable with 3 levels: “current smoker,” “former smoker,” and “never smoker.” We conducted robustness checks of all models with generalized additive models to allow for nonlinear time trends, but in almost all cases, a linear time trend was the best fit for the model (results available from the authors upon request). Since for the small number of cases where there was a nonlinear fit this did not change inference about the patterns or time trends, we present linear trends across all models.

## Results

**Figure 1.**
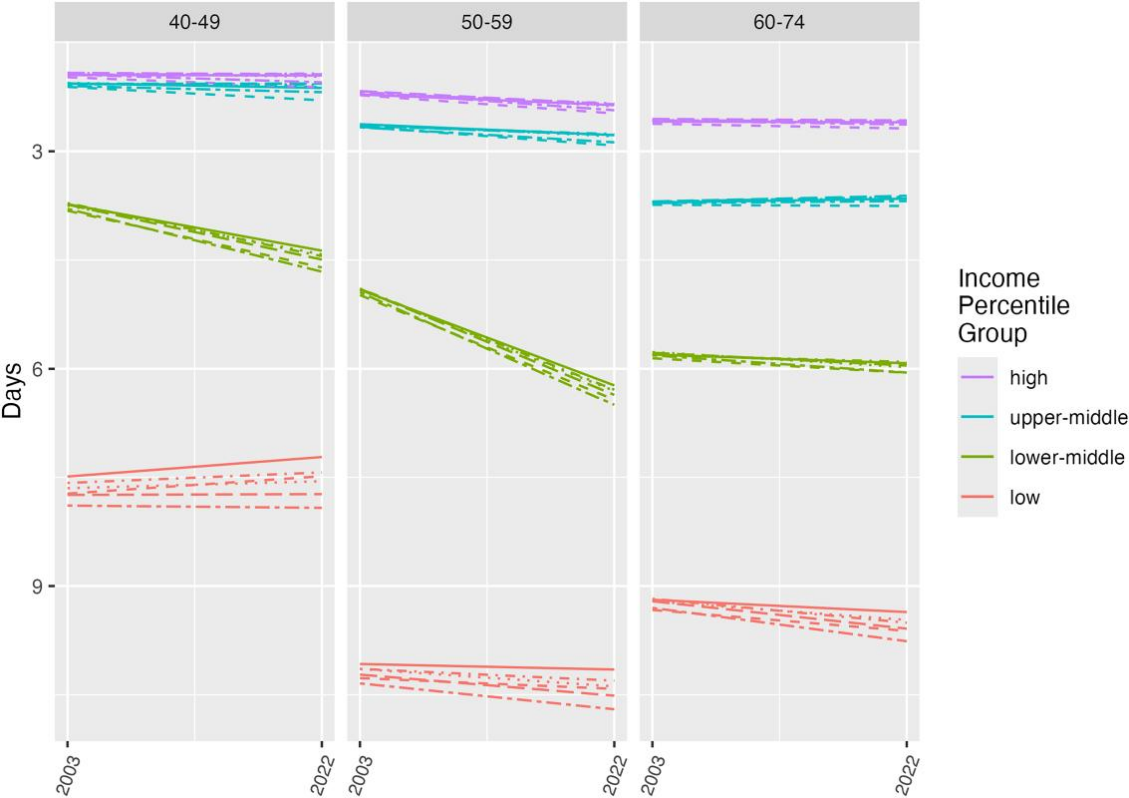
Changes in physical well-being by income level and age: BRFSS, 2003–2022. The lines within each income-category group show results from 6 different regression models: unadjusted/base (solid line); race adjusted (short dashes); BMI adjusted (dots); smoking adjusted (dashes and dots); BMI and smoking adjusted (long dashes); and BMI, smoking, and race and ethnicity adjusted (long dashes and dots). Abbreviations: BMI, body mass index; BRFSS, Behavioral Risk Factor Surveillance System.

**Figure 2.**
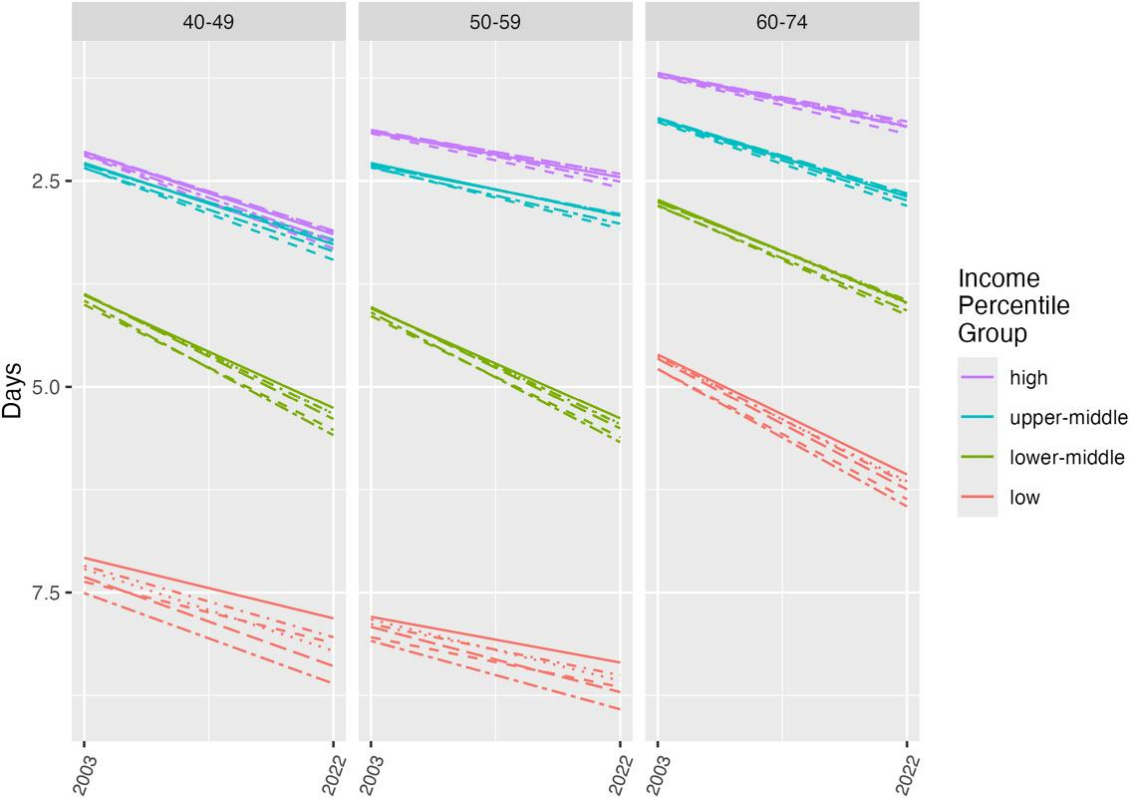
Changes in mental well-being by income level and age: BRFSS, 2003–2022. The lines within each income-category group show results from 6 different regression models: unadjusted/base (solid line); race adjusted (short dashes); BMI adjusted (dots); smoking adjusted (dashes and dots); BMI and smoking adjusted (long dashes); and BMI, smoking, and race and ethnicity adjusted (long dashes and dots). Abbreviations: BMI, body mass index; BRFSS, Behavioral Risk Factor Surveillance System.

**Figure 3.**
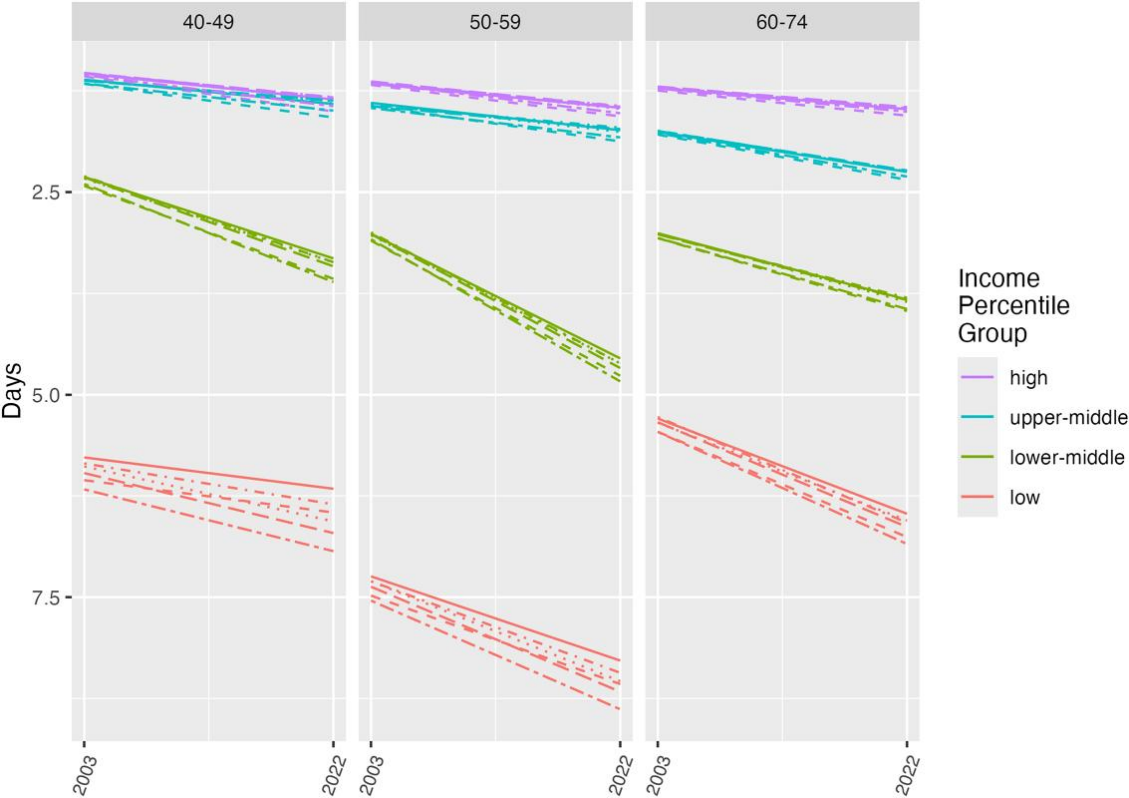
Changes in functional well-being by income level and age: BRFSS, 2003–2022. The lines within each income-category group show results from 6 different regression models: unadjusted/base (solid line); race adjusted (short dashes); BMI adjusted (dots); smoking adjusted (dashes and dot); BMI and smoking adjusted (long dashes); and BMI, smoking, and race and ethnicity adjusted (long dashes and dots). Abbreviations: BMI, body mass index; BRFSS, Behavioral Risk Factor Surveillance System.

Figures 1–3 present our findings for physical well-being, mental well-being, and functional well-being, respectively. Note that the *y*-axis for each figure goes from greater number of days of impairment at the bottom to fewer number of days of impairment at the top, so higher on each plot indicates better well-being. Each of the panels shows the age strata 40–49, 50–59, and 60–74, for the years 2003 to 2022. Income categories specific to each age group are shown in red (low), green (lower-middle), blue (upper-middle), and purple (high), with the focus of our analysis on examining the trends in lower-middle (green lines) and upper-middle (blue lines) time trends. We chose the same age and income groups as were used in recent prior work on the “forgotten middle” in order to facilitate comparison of our findings with that work. The lines within each income category group show results from 6 different regression models: unadjusted/base (solid line); race adjusted (short dashes); BMI adjusted (dots); smoking adjusted (dash and dot); BMI and smoking adjusted (long dashes); and BMI, smoking, and race and ethnicity adjusted (long dash and dot). Regression coefficients for the slope of each line by model can be found in Tables S6–S8 (for physical well-being, mental well-being, and functional well-being, respectively).

Figure 1 presents findings for the number of impaired physical well-being days. We observed strong differences in overall level by income category, where there are 3 or fewer impaired days of well-being per month among the highest-income group (purple lines), even at older ages, as compared with 8 or more impaired physical well-being days per month for those living in the lowest-income households (red lines). Notably, there are substantially worse levels of physical well-being in the lowest-income households for individuals aged 50 to 59, even as compared with older individuals. When looking at changes from 2003 to 2022, there was no change among some income groups and ages, or small declines experienced in days per month of impaired physical well-being for some other income groups. However, we observed by far the most rapid declines in days of impaired physical well-being for the lower-middle-income group (green lines). This decline is particularly notable among individuals ages 50–59, whereby the end of the study period, 2022, physical well-being becomes even worse than the older population aged 60–74 years. The results from all 6 models are similar (as shown by the dashed and dotted lines of the same color within each age and income group), showing that the time trends or differences between income groups are not explained by race and ethnicity, BMI, or smoking.

Figure 2 presents findings for number of impaired mental well-being days per month. As with physical well-being, the magnitude of the differences in impaired mental well-being days per month by household income level is dramatic—on average, 5 more impaired days per month between the highest- and lowest-income groups—and these differences have increased over time in terms of the number of days. In contrast with physical well-being, there were more consistent declines in mental well-being across income categories. For the population aged 50–59 years, however, the number of impaired mental-health days per month becomes larger (worse) for the lower-middle-income population. Notably, the number of bad mental well-being days is worse for the population aged 50 to 59 than for those age 60 to 74 years, at every level of household income. The differences in impaired mental-health days per month also do not appear to be explained by race and ethnicity, BMI, or smoking.

Finally, Figure 3 shows the results for the number of impaired functional well-being days per month. As with physical and mental well-being, we see dramatic differences by household income group, with up to 6 more impaired days per month for individuals in the low-income group. The declines in functional well-being over time are very small for the highest-income group, and large for the lower-middle-income and lowest-household-income groups. For the lower-middle-income population the number of impaired functional well-being days has increased by 2 days per month over this 20-year period. The levels of impaired functional well-being days per month in the lower-middle-income category in the population prior to retirement (ages 50 to 59) are now higher than those in the poorest households at older ages (ages 60 to 74). As with physical and mental well-being, these differences are not explained by race and ethnicity, BMI, or smoking.

While our focus was on using the most up-to-date data available, our interest in examining the longer-term trends in these data brings up the question of the extent to which these trends were driven in part by the recent well-documented declines in well-being that were related to both the COVID-19 pandemic and the responses to the COVID-19 pandemic during 2020, 2021, and 2022. We thus replicated our primary analyses through 2019 and showed that these time trends were not weaker than those including data through 2022, indicating our current findings on trends were not meaningfully impacted by the COVID-19 pandemic (Table S9a–S9c).

We also fit models stratified by race and ethnicity, to examine whether trends differed across racial and ethnic groups including American Indian and Alaska Native persons, Asian persons, Black and African American persons, Latino and Hispanic persons, White persons, and persons who self-identified race or ethnicity in another group that did not fit in 1 of these categories (results shown in Figures S1–S3). Overall, there were few notable differences in time trends, with declines across time in the lower-middle-income group for all populations aged 50–59 years, except among Asian persons for physical and mental well-being, and for Hispanics for mental well-being.

## Discussion

Our descriptive analysis was directed at answering the question of the well-being of populations in the decades prior to retirement, with a focus on these patterns by income categories of low, lower-middle, upper-middle, and high income. We found dramatic and consistent differences in health by income category. While there was worsening well-being over the last 20 years for all ages, there were the most dramatic declines for the poorest and lower-middle groups, with declining trends being greatest for lower-middle-income households. No trends or levels were explained by race and ethnicity, BMI, or smoking. It is notable that these trends were not specific to 1 domain or type of outcome but were consistent across 3 different measures of well-being—physical, mental, and functional—but were least different for mental well-being.

The trends that we show, which are steady and very consistent across the past 20 years, and which we show were occurring prior to the COVID-19 pandemic, have troubling implications for the health of the population soon to be entering retirement. Our findings dispute the optimistic scenario of the compression of morbidity, at least for the “forgotten middle.” If current trends persist, the current 50–59-year-old population will be entering retirement ages with substantially worse health than previous generations. Our findings build on the prior work by Chapel et al,^7^ who used a different dataset and methods of projection based on a microsimulation model. The consistency of these findings suggests that these trends are real, and that we need to better understand the drivers of these trends and develop approaches to promoting general well-being and health not only in the lowest-income households but also in households in the lower-middle-income group, who are not generally eligible for most social benefit programs. It is particularly notable that the 1 exception to this, the Earned Income Tax Credit, only benefits households with adults who can work and have dependent children. Given that many individuals aged 50–59 years no longer have dependent children in the household, and that the number of impaired physical- and mental-health days may prohibit employment, even the Earned Income Tax Credit is not likely to benefit most of these households. In addition, we found that these declines in well-being were not impacted by the COVID-19 pandemic and responses. This finding is in line with other work that showed little change in depression due to COVID-19 among these ages, but instead, steady increases over time.^16^

Another notable finding is that the health gradients are much larger in the 50- to 59-year-old population than in the population aged 60 to 74 years for mental and functional health. This is not likely due to mortality selection or due to the relevance of income at different ages, because this trend does not hold as strongly for physical health. Future work should investigate these specific gradients more closely to uncover the reasons for this, including the extent to which access to medical care through Medicare and income supports through Social Security payments have protected these older populations from the downward trends impacting the pre-retirement population.^17^

Our findings are comparable but unique to several other studies documenting health trends in the United States over time. Like work on the “deaths of despair,” our findings note rapid declines in health for lower-income individuals.^18,19^ Unlike these findings, however, we noted that the well-being declines include a much larger portion of the population, and are most dramatic among lower-middle-income populations. A further contrast with the general trends of this work is that our work shows that all racial and ethnic groups are impacted by these trends equally, with the exception of individuals self-identifying as Asian. In addition, as compared with other recent studies of health declines in the United States over several decades,^20^ our work takes a particular focus on the population that will be entering retirement ages over the next decade, which has specific implications for policies related to health and benefits for persons exiting the labor force.

There are several limitations to our analysis. As described in the Data and methods section, we were limited in the number of outcomes that we could examine, and there are not more detailed functional, clinical, or biomarker-based measures that could give a more nuanced view of morbidity.^9,21^ Even though these 3 measures are self-reported and general, they are well validated and capture important aspects of the impact of morbidity on social engagement and functioning. A second limitation is that we do not have measures of household wealth or debt, so can only capture income as resources for households. A third limitation is that with cross-sectional data it is difficult to account for mortality selection, so differences in the prevalence we observed, in the 60- to 74-year age category, are in part driven by this. This is unlikely to account for any meaningful proportion of the time trends we observe prior to age 60. In addition, since our focus here is on the burden of morbidity in the population, we wanted to capture that burden as it is and not adjust this burden by statistical methods used in etiologic research that need to adjust for mortality selection. Finally, we were not able to match percentiles of income exactly to prior work (as shown in Table S5). There were no marked temporal differences in the proportion of individuals in each category, however, so any small differences in the proportion of individuals by income category are not expected to be driving the main trends we observed over time in the income groups that we focused on.

## Conclusion

In summary, we found strong 20-year declining trends in 3 different measures of well-being for the lower-middle-income population—the group where household income is between the 15th and 45th percentiles of the income distribution. The strongest downward trends are among the population aged 50 to 59, as compared with the population age 40 to 49 and the population aged 60 to 74. It is notable that, over time, our measures of well-being have declined to such a large extent in the population aged 50 to 59 that they now have worse levels of health-related well-being than the older population. While declines in well-being for this age and income group are enough on their own to make them worthy of policy attention, given the fact that these trends are specific to the population just prior to retirement age and for a group less likely to have retirement savings, there are also additional important policy implications for late-middle-life and old-age labor force participation, as well as for Medicare and Social Security.

## Acknowledgments

The members of the Research Network on an Aging Society include John W. Rowe (chair), Toni C. Antonucci, Lisa Berkman, Axel Börsch-Supan, Laura L. Carstensen, Cynthia Chen, Dana P. Goldman, Linda P. Fried, Frank F. Furstenberg, Martin Kohli, S. Jay Olshansky, David H. Rehkopf, and Julie Zissimopoulos.

## Supplementary Material

qxae183_Supplementary_Data

## Data Availability

All data used for this analysis is publically available at https://www.cdc.gov/brfss/index.html. All code for the analysis is available from the corresponding author upon request.
