# Supplementary material for: Relation of income to trends in well-being by age: implications for the future older “forgotten” lower middle class
Source: Health Aff Sch. 2025 Jan 9;3(2):qxae183. doi: 10.1093/haschl/qxae183 (PMC11829254; doi:10.1093/haschl/qxae183)

**Supplemental Tables and Figures**

**Table S1** Equivalized income missing data by year, BRFSS 2003 – 2022, ages 40-74

| **year** | **equivalized income**  **missing data** | |
| --- | --- | --- |
|  | **count** | **percent** |
| 2003 | 19,144 | 0.13 |
| 2004 | 22,274 | 0.13 |
| 2005 | 26,724 | 0.12 |
| 2006 | 27,412 | 0.12 |
| 2007 | 32,852 | 0.12 |
| 2008 | 29,744 | 0.11 |
| 2009 | 32,581 | 0.12 |
| 2010 | 36,319 | 0.12 |
| 2011 | 39,596 | 0.12 |
| 2012 | 35,228 | 0.12 |
| 2013 | 38,113 | 0.12 |
| 2014 | 39,559 | 0.14 |
| 2015 | 44,533 | 0.16 |
| 2016 | 45,703 | 0.15 |
| 2017 | 58,342 | 0.21 |
| 2018 | 38,564 | 0.15 |
| 2019 | 41,132 | 0.17 |
| 2020 | 40,605 | 0.17 |
| 2021 | 48,786 | 0.19 |
| 2022 | 48,753 | 0.19 |

**Table S2** Missing data by outcome and model, BRFSS 2003 – 2022, ages 40-74

| **outcome** | **model** | **missing count** | **missing percent** |
| --- | --- | --- | --- |
| physical | base | 827,879 | 15.6 |
| physical | race | 873,629 | 16.5 |
| physical | BMI | 1,002,971 | 18.9 |
| physical | smoke | 894,040 | 16.8 |
| physical | BMI + smoke | 1,054,316 | 19.9 |
| physical | BMI + smoke + race | 1,094,927 | 20.6 |
| mental | base | 817,460 | 15.4 |
| mental | race | 863,296 | 16.3 |
| mental | BMI | 993,264 | 18.7 |
| mental | smoke | 883,868 | 16.7 |
| mental | BMI + smoke | 1,044,740 | 19.7 |
| mental | BMI + smoke + race | 1,085,401 | 20.4 |
| functional | base | 797,414 | 15.0 |
| functional | race | 843,783 | 15.9 |
| functional | BMI | 974,802 | 18.4 |
| functional | smoke | 864,405 | 16.3 |
| functional | BMI + smoke | 1,026,762 | 19.3 |
| functional | BMI + smoke + race | 1,067,902 | 20.1 |

| **Table S3** BRFSS income categories, BRFSS 2003 – 2022, ages 40-74 | | | | |
| --- | --- | --- | --- | --- |
| **BRFSS code** | **analytic recode** | **INCOME2**  **(2003-2020)** | **INCOME3**  **(2021-2022)** |  |
| 1 | 1 | $0 - $10k | $0 - $10k |  |
| 2 | 2 | $10k - $15k | $10k - $15k |  |
| 3 | 3 | $15k - $20k | $15k - $20k |  |
| 4 | 4 | $20k - $25k | $20k - $25k |  |
| 5 | 5 | $25k - $35k | $25k - $35k |  |
| 6 | 6 | $35k - $50k | $35k - $50k |  |
| 7 | 7 | $50k - $75k | $50k - $75k |  |
| 8 | 8 | > $75k | $75k - $100k |  |
| 9 | 8 | NA | $100k - $150k |  |
| 10 | 8 | NA | $150k - $200k |  |
| 11 | 8 | NA | > $200k |  |

Table notes: BRFSS provides a categorical variable, INCOME2, that is available for all survey years from 2003 – 2020. Beginning in 2021 INCOME2 is updated with three additional upper-income categories and renamed INCOME3.

| **Table S4** Distribution of population across income category, BRFSS 2003 – 2022, ages 40-74 | | |
| --- | --- | --- |
| **income group** | **target (Chapel et al.)** | **BRFSS coverage**  **mean (95% CI)** |
| Low | 0.15 | 0.160 (0.157, 0.162) |
| Lower Middle | 0.30 | 0.312 (0.306, 0.318) |
| Upper Middle | 0.30 | 0.324 (0.314, 0.334) |
| High | 0.25 | 0.204 (0.196, 0.212) |

Table notes: On average, we achieved a distribution that was within a few percentage points of the target category proportion

| **Table S5** Coverage across income categories by year and age, BRFSS 2003 – 2022, ages 40-74 | | | | | | | | | | |
| --- | --- | --- | --- | --- | --- | --- | --- | --- | --- | --- |
| **year** | **age** | | **low**  **(15%)** | | **lower middle (30%)** | | **upper middle (30%)** | | **upper**  **(25%)** | |
| 2003 | 40-49 | | 0.15 | | 0.31 | | 0.29 | | 0.25 | |
| 2004 | 40-49 | | 0.15 | | 0.31 | | 0.39 | | 0.15 | |
| 2005 | 40-49 | | 0.15 | | 0.33 | | 0.36 | | 0.16 | |
| 2006 | 40-49 | | 0.16 | | 0.34 | | 0.34 | | 0.16 | |
| 2007 | 40-49 | | 0.15 | | 0.34 | | 0.34 | | 0.17 | |
| 2008 | 40-49 | | 0.15 | | 0.33 | | 0.35 | | 0.17 | |
| 2009 | 40-49 | | 0.15 | | 0.33 | | 0.36 | | 0.16 | |
| 2010 | 40-49 | | 0.15 | | 0.32 | | 0.36 | | 0.17 | |
| 2011 | 40-49 | | 0.16 | | 0.30 | | 0.39 | | 0.16 | |
| 2012 | 40-49 | | 0.15 | | 0.32 | | 0.37 | | 0.17 | |
| 2013 | 40-49 | | 0.15 | | 0.33 | | 0.35 | | 0.18 | |
| 2014 | 40-49 | | 0.15 | | 0.32 | | 0.36 | | 0.17 | |
| 2015 | 40-49 | | 0.16 | | 0.29 | | 0.37 | | 0.18 | |
| 2016 | 40-49 | | 0.15 | | 0.32 | | 0.34 | | 0.19 | |
| 2017 | 40-49 | | 0.16 | | 0.30 | | 0.33 | | 0.21 | |
| 2018 | 40-49 | | 0.15 | | 0.32 | | 0.35 | | 0.18 | |
| 2019 | 40-49 | | 0.15 | | 0.32 | | 0.34 | | 0.19 | |
| 2020 | 40-49 | | 0.15 | | 0.31 | | 0.34 | | 0.20 | |
| 2021 | 40-49 | | 0.15 | | 0.30 | | 0.31 | | 0.24 | |
| 2022 | 40-49 | | 0.16 | | 0.29 | | 0.30 | | 0.25 | |
| 2003 | 50-59 | | 0.16 | | 0.33 | | 0.28 | | 0.24 | |
| 2004 | 50-59 | | 0.16 | | 0.32 | | 0.28 | | 0.24 | |
| 2005 | 50-59 | | 0.16 | | 0.30 | | 0.37 | | 0.18 | |
| 2006 | 50-59 | | 0.15 | | 0.30 | | 0.36 | | 0.19 | |
| 2007 | 50-59 | | 0.15 | | 0.33 | | 0.33 | | 0.19 | |
| 2008 | 50-59 | | 0.16 | | 0.33 | | 0.32 | | 0.20 | |
| 2009 | 50-59 | | 0.15 | | 0.34 | | 0.32 | | 0.19 | |
| 2010 | 50-59 | | 0.16 | | 0.29 | | 0.36 | | 0.18 | |
| 2011 | 50-59 | | 0.16 | | 0.31 | | 0.35 | | 0.18 | |
| 2012 | 50-59 | | 0.16 | | 0.30 | | 0.35 | | 0.19 | |
| 2013 | 50-59 | | 0.17 | | 0.30 | | 0.34 | | 0.20 | |
| 2014 | 50-59 | | 0.15 | | 0.31 | | 0.36 | | 0.18 | |
| 2015 | 50-59 | | 0.17 | | 0.30 | | 0.35 | | 0.18 | |
| 2016 | 50-59 | | 0.16 | | 0.29 | | 0.35 | | 0.19 | |
| 2017 | 50-59 | | 0.16 | | 0.29 | | 0.34 | | 0.21 | |
| 2018 | 50-59 | | 0.16 | | 0.31 | | 0.34 | | 0.19 | |
| 2019 | 50-59 | | 0.16 | | 0.30 | | 0.34 | | 0.20 | |
| 2020 | 50-59 | | 0.15 | | 0.30 | | 0.34 | | 0.21 | |
| 2021 | 50-59 | | 0.16 | | 0.31 | | 0.31 | | 0.23 | |
| 2022 | 50-59 | | 0.15 | | 0.34 | | 0.31 | | 0.19 | |
| 2003 | 60-74 | | 0.19 | | 0.27 | | 0.36 | | 0.17 | |
| 2004 | 60-74 | | 0.18 | | 0.27 | | 0.37 | | 0.18 | |
| 2005 | 60-74 | | 0.18 | | 0.30 | | 0.33 | | 0.19 | |
| 2006 | 60-74 | | 0.16 | | 0.30 | | 0.31 | | 0.23 | |
| 2007 | 60-74 | | 0.15 | | 0.32 | | 0.30 | | 0.23 | |
| 2008 | 60-74 | | 0.17 | | 0.28 | | 0.31 | | 0.24 | |
| 2009 | 60-74 | | 0.17 | | 0.29 | | 0.31 | | 0.24 | |
| 2010 | 60-74 | | 0.17 | | 0.38 | | 0.20 | | 0.25 | |
| 2011 | 60-74 | | 0.15 | | 0.33 | | 0.27 | | 0.25 | |
| 2012 | 60-74 | | 0.17 | | 0.28 | | 0.31 | | 0.24 | |
| 2013 | 60-74 | | 0.17 | | 0.28 | | 0.30 | | 0.25 | |
| 2014 | 60-74 | | 0.18 | | 0.36 | | 0.23 | | 0.24 | |
| 2015 | 60-74 | | 0.17 | | 0.36 | | 0.26 | | 0.22 | |
| 2016 | 60-74 | | 0.17 | | 0.34 | | 0.27 | | 0.22 | |
| 2017 | 60-74 | | 0.16 | | 0.33 | | 0.26 | | 0.24 | |
| 2018 | 60-74 | | 0.16 | | 0.32 | | 0.28 | | 0.24 | |
| 2019 | 60-74 | | 0.16 | | 0.32 | | 0.27 | | 0.25 | |
| 2020 | 60-74 | | 0.16 | | 0.31 | | 0.28 | | 0.25 | |
| 2021 | 60-74 | | 0.17 | | 0.28 | | 0.33 | | 0.22 | |
| 2022 | 60-74 | | 0.16 | | 0.31 | | 0.29 | | 0.24 | |
| **Mean (95% CI)** | | 0.160 (0.157, 0.162) | | 0.312 (0.306, 0.318) | | 0.324 (0.314, 0.334) | | 0.204 (0.196, 0.212) | |  |

**Table S6** Linear time trend slopes by model, physical well-being, BRFSS 2003 – 2022, ages 40-74

| **age** | **income** | **model** | **slope** | **95% CI** |
| --- | --- | --- | --- | --- |
| 40-49 | 0-15 | base | -0.01 (p = 0.0000) | [-0.02, -0.01] |
| 40-49 | 0-15 | base + race | -0.01 (p = 0.1029) | [-0.01, 0.00] |
| 40-49 | 0-15 | base + BMI | -0.02 (p = 0.0000) | [-0.03, -0.02] |
| 40-49 | 0-15 | base + smoke | 0.00 (p = 0.2802) | [-0.00, 0.01] |
| 40-49 | 0-15 | base + BMI + smoke | -0.01 (p = 0.0190) | [-0.01, -0.00] |
| 40-49 | 0-15 | base + BMI + smoke + race | -0.00 (p = 0.3627) | [-0.01, 0.00] |
| 40-49 | 16-45 | base | 0.03 (p = 0.0000) | [0.03, 0.04] |
| 40-49 | 16-45 | base + race | 0.05 (p = 0.0000) | [0.04, 0.05] |
| 40-49 | 16-45 | base + BMI | 0.02 (p = 0.0000) | [0.01, 0.02] |
| 40-49 | 16-45 | base + smoke | 0.04 (p = 0.0000) | [0.04, 0.05] |
| 40-49 | 16-45 | base + BMI + smoke | 0.02 (p = 0.0000) | [0.02, 0.03] |
| 40-49 | 16-45 | base + BMI + smoke + race | 0.03 (p = 0.0000) | [0.03, 0.04] |
| 40-49 | 46-75 | base | 0.00 (p = 0.1197) | [-0.00, 0.01] |
| 40-49 | 46-75 | base + race | 0.01 (p = 0.0000) | [0.01, 0.02] |
| 40-49 | 46-75 | base + BMI | -0.01 (p = 0.0000) | [-0.02, -0.01] |
| 40-49 | 46-75 | base + smoke | 0.01 (p = 0.0000) | [0.01, 0.01] |
| 40-49 | 46-75 | base + BMI + smoke | -0.01 (p = 0.0010) | [-0.01, -0.00] |
| 40-49 | 46-75 | base + BMI + smoke + race | -0.00 (p = 0.5271) | [-0.01, 0.00] |
| 40-49 | 76-100 | base | 0.00 (p = 0.8751) | [-0.00, 0.01] |
| 40-49 | 76-100 | base + race | 0.01 (p = 0.0000) | [0.01, 0.02] |
| 40-49 | 76-100 | base + BMI | -0.01 (p = 0.0000) | [-0.02, -0.01] |
| 40-49 | 76-100 | base + smoke | 0.01 (p = 0.0089) | [0.00, 0.01] |
| 40-49 | 76-100 | base + BMI + smoke | -0.01 (p = 0.0162) | [-0.01, -0.00] |
| 40-49 | 76-100 | base + BMI + smoke + race | -0.00 (p = 0.9826) | [-0.01, 0.01] |
| 50-59 | 0-15 | base | 0.00 (p = 0.2161) | [-0.00, 0.01] |
| 50-59 | 0-15 | base + race | 0.01 (p = 0.0022) | [0.00, 0.02] |
| 50-59 | 0-15 | base + BMI | 0.00 (p = 0.3879) | [-0.00, 0.01] |
| 50-59 | 0-15 | base + smoke | 0.01 (p = 0.0007) | [0.00, 0.02] |
| 50-59 | 0-15 | base + BMI + smoke | 0.01 (p = 0.0305) | [0.00, 0.01] |
| 50-59 | 0-15 | base + BMI + smoke + race | 0.01 (p = 0.0018) | [0.00, 0.02] |
| 50-59 | 16-45 | base | 0.07 (p = 0.0000) | [0.06, 0.07] |
| 50-59 | 16-45 | base + race | 0.08 (p = 0.0000) | [0.08, 0.08] |
| 50-59 | 16-45 | base + BMI | 0.06 (p = 0.0000) | [0.05, 0.06] |
| 50-59 | 16-45 | base + smoke | 0.08 (p = 0.0000) | [0.07, 0.08] |
| 50-59 | 16-45 | base + BMI + smoke | 0.07 (p = 0.0000) | [0.06, 0.07] |
| 50-59 | 16-45 | base + BMI + smoke + race | 0.07 (p = 0.0000) | [0.07, 0.08] |
| 50-59 | 46-75 | base | 0.01 (p = 0.0004) | [0.00, 0.01] |
| 50-59 | 46-75 | base + race | 0.02 (p = 0.0000) | [0.01, 0.02] |
| 50-59 | 46-75 | base + BMI | -0.00 (p = 0.0461) | [-0.01, -0.00] |
| 50-59 | 46-75 | base + smoke | 0.02 (p = 0.0000) | [0.02, 0.02] |
| 50-59 | 46-75 | base + BMI + smoke | 0.01 (p = 0.0021) | [0.00, 0.01] |
| 50-59 | 46-75 | base + BMI + smoke + race | 0.01 (p = 0.0000) | [0.01, 0.02] |
| 50-59 | 76-100 | base | 0.01 (p = 0.0050) | [0.00, 0.01] |
| 50-59 | 76-100 | base + race | 0.02 (p = 0.0000) | [0.01, 0.02] |
| 50-59 | 76-100 | base + BMI | -0.00 (p = 0.5897) | [-0.01, 0.00] |
| 50-59 | 76-100 | base + smoke | 0.02 (p = 0.0000) | [0.02, 0.03] |
| 50-59 | 76-100 | base + BMI + smoke | 0.01 (p = 0.0001) | [0.01, 0.02] |
| 50-59 | 76-100 | base + BMI + smoke + race | 0.02 (p = 0.0000) | [0.01, 0.02] |
| 60-74 | 0-15 | base | 0.01 (p = 0.0041) | [0.00, 0.02] |
| 60-74 | 0-15 | base + race | 0.01 (p = 0.0000) | [0.01, 0.02] |
| 60-74 | 0-15 | base + BMI | 0.00 (p = 0.3923) | [-0.00, 0.01] |
| 60-74 | 0-15 | base + smoke | 0.01 (p = 0.0000) | [0.01, 0.02] |
| 60-74 | 0-15 | base + BMI + smoke | 0.00 (p = 0.2535) | [-0.00, 0.01] |
| 60-74 | 0-15 | base + BMI + smoke + race | 0.01 (p = 0.0265) | [0.00, 0.01] |
| 60-74 | 16-45 | base | 0.01 (p = 0.0063) | [0.00, 0.01] |
| 60-74 | 16-45 | base + race | 0.01 (p = 0.0000) | [0.01, 0.02] |
| 60-74 | 16-45 | base + BMI | -0.00 (p = 0.6321) | [-0.01, 0.00] |
| 60-74 | 16-45 | base + smoke | 0.01 (p = 0.0000) | [0.01, 0.01] |
| 60-74 | 16-45 | base + BMI + smoke | 0.00 (p = 0.5236) | [-0.00, 0.01] |
| 60-74 | 16-45 | base + BMI + smoke + race | 0.00 (p = 0.0444) | [0.00, 0.01] |
| 60-74 | 46-75 | base | -0.00 (p = 0.2046) | [-0.01, 0.00] |
| 60-74 | 46-75 | base + race | 0.00 (p = 0.3924) | [-0.00, 0.01] |
| 60-74 | 46-75 | base + BMI | -0.02 (p = 0.0000) | [-0.02, -0.01] |
| 60-74 | 46-75 | base + smoke | 0.01 (p = 0.0003) | [0.00, 0.01] |
| 60-74 | 46-75 | base + BMI + smoke | -0.01 (p = 0.0168) | [-0.01, -0.00] |
| 60-74 | 46-75 | base + BMI + smoke + race | -0.00 (p = 0.2453) | [-0.01, 0.00] |
| 60-74 | 76-100 | base | 0.00 (p = 0.8530) | [-0.00, 0.01] |
| 60-74 | 76-100 | base + race | 0.00 (p = 0.1315) | [-0.00, 0.01] |
| 60-74 | 76-100 | base + BMI | -0.01 (p = 0.0008) | [-0.01, -0.00] |
| 60-74 | 76-100 | base + smoke | 0.02 (p = 0.0000) | [0.01, 0.02] |
| 60-74 | 76-100 | base + BMI + smoke | 0.01 (p = 0.0436) | [0.00, 0.01] |
| 60-74 | 76-100 | base + BMI + smoke + race | 0.01 (p = 0.0104) | [0.00, 0.01] |

**Table S7** Linear time trend slopes by model, mental well-being, BRFSS 2003 – 2022, ages 40-74

| **age** | **income** | **model** | **slope** | **95% CI** |
| --- | --- | --- | --- | --- |
| 40-49 | 0-15 | base | 0.04 (p = 0.0000) | [0.03, 0.04] |
| 40-49 | 0-15 | base + race | 0.05 (p = 0.0000) | [0.05, 0.06] |
| 40-49 | 0-15 | base + BMI | 0.04 (p = 0.0000) | [0.04, 0.05] |
| 40-49 | 0-15 | base + smoke | 0.06 (p = 0.0000) | [0.05, 0.07] |
| 40-49 | 0-15 | base + BMI + smoke | 0.06 (p = 0.0000) | [0.05, 0.07] |
| 40-49 | 0-15 | base + BMI + smoke + race | 0.07 (p = 0.0000) | [0.06, 0.07] |
| 40-49 | 16-45 | base | 0.07 (p = 0.0000) | [0.07, 0.08] |
| 40-49 | 16-45 | base + race | 0.09 (p = 0.0000) | [0.09, 0.09] |
| 40-49 | 16-45 | base + BMI | 0.07 (p = 0.0000) | [0.06, 0.07] |
| 40-49 | 16-45 | base + smoke | 0.08 (p = 0.0000) | [0.08, 0.09] |
| 40-49 | 16-45 | base + BMI + smoke | 0.08 (p = 0.0000) | [0.07, 0.08] |
| 40-49 | 16-45 | base + BMI + smoke + race | 0.09 (p = 0.0000) | [0.08, 0.09] |
| 40-49 | 46-75 | base | 0.05 (p = 0.0000) | [0.05, 0.06] |
| 40-49 | 46-75 | base + race | 0.06 (p = 0.0000) | [0.06, 0.07] |
| 40-49 | 46-75 | base + BMI | 0.04 (p = 0.0000) | [0.04, 0.05] |
| 40-49 | 46-75 | base + smoke | 0.06 (p = 0.0000) | [0.06, 0.06] |
| 40-49 | 46-75 | base + BMI + smoke | 0.05 (p = 0.0000) | [0.05, 0.06] |
| 40-49 | 46-75 | base + BMI + smoke + race | 0.06 (p = 0.0000) | [0.05, 0.06] |
| 40-49 | 76-100 | base | 0.05 (p = 0.0000) | [0.05, 0.06] |
| 40-49 | 76-100 | base + race | 0.06 (p = 0.0000) | [0.06, 0.07] |
| 40-49 | 76-100 | base + BMI | 0.05 (p = 0.0000) | [0.04, 0.05] |
| 40-49 | 76-100 | base + smoke | 0.06 (p = 0.0000) | [0.06, 0.07] |
| 40-49 | 76-100 | base + BMI + smoke | 0.05 (p = 0.0000) | [0.05, 0.06] |
| 40-49 | 76-100 | base + BMI + smoke + race | 0.06 (p = 0.0000) | [0.06, 0.07] |
| 50-59 | 0-15 | base | 0.03 (p = 0.0000) | [0.02, 0.04] |
| 50-59 | 0-15 | base + race | 0.04 (p = 0.0000) | [0.03, 0.04] |
| 50-59 | 0-15 | base + BMI | 0.03 (p = 0.0000) | [0.03, 0.04] |
| 50-59 | 0-15 | base + smoke | 0.04 (p = 0.0000) | [0.03, 0.04] |
| 50-59 | 0-15 | base + BMI + smoke | 0.04 (p = 0.0000) | [0.03, 0.04] |
| 50-59 | 0-15 | base + BMI + smoke + race | 0.04 (p = 0.0000) | [0.04, 0.05] |
| 50-59 | 16-45 | base | 0.07 (p = 0.0000) | [0.07, 0.07] |
| 50-59 | 16-45 | base + race | 0.08 (p = 0.0000) | [0.08, 0.09] |
| 50-59 | 16-45 | base + BMI | 0.07 (p = 0.0000) | [0.06, 0.07] |
| 50-59 | 16-45 | base + smoke | 0.08 (p = 0.0000) | [0.08, 0.08] |
| 50-59 | 16-45 | base + BMI + smoke | 0.07 (p = 0.0000) | [0.07, 0.08] |
| 50-59 | 16-45 | base + BMI + smoke + race | 0.08 (p = 0.0000) | [0.08, 0.09] |
| 50-59 | 46-75 | base | 0.03 (p = 0.0000) | [0.03, 0.04] |
| 50-59 | 46-75 | base + race | 0.04 (p = 0.0000) | [0.04, 0.05] |
| 50-59 | 46-75 | base + BMI | 0.03 (p = 0.0000) | [0.02, 0.03] |
| 50-59 | 46-75 | base + smoke | 0.05 (p = 0.0000) | [0.04, 0.05] |
| 50-59 | 46-75 | base + BMI + smoke | 0.04 (p = 0.0000) | [0.03, 0.04] |
| 50-59 | 46-75 | base + BMI + smoke + race | 0.04 (p = 0.0000) | [0.04, 0.05] |
| 50-59 | 76-100 | base | 0.03 (p = 0.0000) | [0.02, 0.03] |
| 50-59 | 76-100 | base + race | 0.04 (p = 0.0000) | [0.03, 0.04] |
| 50-59 | 76-100 | base + BMI | 0.02 (p = 0.0000) | [0.02, 0.03] |
| 50-59 | 76-100 | base + smoke | 0.04 (p = 0.0000) | [0.04, 0.05] |
| 50-59 | 76-100 | base + BMI + smoke | 0.04 (p = 0.0000) | [0.03, 0.04] |
| 50-59 | 76-100 | base + BMI + smoke + race | 0.04 (p = 0.0000) | [0.04, 0.05] |
| 60-74 | 0-15 | base | 0.08 (p = 0.0000) | [0.07, 0.08] |
| 60-74 | 0-15 | base + race | 0.08 (p = 0.0000) | [0.08, 0.09] |
| 60-74 | 0-15 | base + BMI | 0.07 (p = 0.0000) | [0.07, 0.08] |
| 60-74 | 0-15 | base + smoke | 0.08 (p = 0.0000) | [0.07, 0.08] |
| 60-74 | 0-15 | base + BMI + smoke | 0.07 (p = 0.0000) | [0.06, 0.08] |
| 60-74 | 0-15 | base + BMI + smoke + race | 0.07 (p = 0.0000) | [0.07, 0.08] |
| 60-74 | 16-45 | base | 0.07 (p = 0.0000) | [0.06, 0.07] |
| 60-74 | 16-45 | base + race | 0.07 (p = 0.0000) | [0.07, 0.08] |
| 60-74 | 16-45 | base + BMI | 0.06 (p = 0.0000) | [0.06, 0.06] |
| 60-74 | 16-45 | base + smoke | 0.07 (p = 0.0000) | [0.06, 0.07] |
| 60-74 | 16-45 | base + BMI + smoke | 0.06 (p = 0.0000) | [0.06, 0.06] |
| 60-74 | 16-45 | base + BMI + smoke + race | 0.06 (p = 0.0000) | [0.06, 0.07] |
| 60-74 | 46-75 | base | 0.05 (p = 0.0000) | [0.05, 0.05] |
| 60-74 | 46-75 | base + race | 0.05 (p = 0.0000) | [0.05, 0.06] |
| 60-74 | 46-75 | base + BMI | 0.04 (p = 0.0000) | [0.04, 0.05] |
| 60-74 | 46-75 | base + smoke | 0.06 (p = 0.0000) | [0.06, 0.06] |
| 60-74 | 46-75 | base + BMI + smoke | 0.05 (p = 0.0000) | [0.05, 0.06] |
| 60-74 | 46-75 | base + BMI + smoke + race | 0.06 (p = 0.0000) | [0.05, 0.06] |
| 60-74 | 76-100 | base | 0.03 (p = 0.0000) | [0.03, 0.04] |
| 60-74 | 76-100 | base + race | 0.04 (p = 0.0000) | [0.03, 0.04] |
| 60-74 | 76-100 | base + BMI | 0.03 (p = 0.0000) | [0.02, 0.03] |
| 60-74 | 76-100 | base + smoke | 0.05 (p = 0.0000) | [0.04, 0.05] |
| 60-74 | 76-100 | base + BMI + smoke | 0.04 (p = 0.0000) | [0.03, 0.04] |
| 60-74 | 76-100 | base + BMI + smoke + race | 0.04 (p = 0.0000) | [0.04, 0.04] |

**Table S8** Linear time trend slopes by model, functional well-being, BRFSS 2003 – 2022, ages 40-74

| **age** | **income** | **model** | **slope** | **95% CI** |
| --- | --- | --- | --- | --- |
| 40-49 | 0-15 | base | 0.02 (p = 0.0000) | [0.02, 0.03] |
| 40-49 | 0-15 | base + race | 0.03 (p = 0.0000) | [0.03, 0.04] |
| 40-49 | 0-15 | base + BMI | 0.02 (p = 0.0000) | [0.02, 0.03] |
| 40-49 | 0-15 | base + smoke | 0.04 (p = 0.0000) | [0.03, 0.04] |
| 40-49 | 0-15 | base + BMI + smoke | 0.04 (p = 0.0000) | [0.03, 0.04] |
| 40-49 | 0-15 | base + BMI + smoke + race | 0.04 (p = 0.0000) | [0.04, 0.05] |
| 40-49 | 16-45 | base | 0.05 (p = 0.0000) | [0.05, 0.06] |
| 40-49 | 16-45 | base + race | 0.07 (p = 0.0000) | [0.07, 0.07] |
| 40-49 | 16-45 | base + BMI | 0.04 (p = 0.0000) | [0.04, 0.04] |
| 40-49 | 16-45 | base + smoke | 0.06 (p = 0.0000) | [0.06, 0.06] |
| 40-49 | 16-45 | base + BMI + smoke | 0.05 (p = 0.0000) | [0.05, 0.05] |
| 40-49 | 16-45 | base + BMI + smoke + race | 0.06 (p = 0.0000) | [0.06, 0.06] |
| 40-49 | 46-75 | base | 0.02 (p = 0.0000) | [0.01, 0.02] |
| 40-49 | 46-75 | base + race | 0.03 (p = 0.0000) | [0.02, 0.03] |
| 40-49 | 46-75 | base + BMI | 0.01 (p = 0.0028) | [0.00, 0.01] |
| 40-49 | 46-75 | base + smoke | 0.02 (p = 0.0000) | [0.02, 0.03] |
| 40-49 | 46-75 | base + BMI + smoke | 0.01 (p = 0.0000) | [0.01, 0.01] |
| 40-49 | 46-75 | base + BMI + smoke + race | 0.02 (p = 0.0000) | [0.01, 0.02] |
| 40-49 | 76-100 | base | 0.02 (p = 0.0000) | [0.01, 0.02] |
| 40-49 | 76-100 | base + race | 0.03 (p = 0.0000) | [0.02, 0.03] |
| 40-49 | 76-100 | base + BMI | 0.01 (p = 0.0004) | [0.00, 0.01] |
| 40-49 | 76-100 | base + smoke | 0.02 (p = 0.0000) | [0.02, 0.03] |
| 40-49 | 76-100 | base + BMI + smoke | 0.01 (p = 0.0000) | [0.01, 0.02] |
| 40-49 | 76-100 | base + BMI + smoke + race | 0.02 (p = 0.0000) | [0.01, 0.02] |
| 50-59 | 0-15 | base | 0.05 (p = 0.0000) | [0.05, 0.06] |
| 50-59 | 0-15 | base + race | 0.06 (p = 0.0000) | [0.06, 0.07] |
| 50-59 | 0-15 | base + BMI | 0.06 (p = 0.0000) | [0.05, 0.06] |
| 50-59 | 0-15 | base + smoke | 0.06 (p = 0.0000) | [0.06, 0.07] |
| 50-59 | 0-15 | base + BMI + smoke | 0.06 (p = 0.0000) | [0.06, 0.07] |
| 50-59 | 0-15 | base + BMI + smoke + race | 0.07 (p = 0.0000) | [0.06, 0.07] |
| 50-59 | 16-45 | base | 0.08 (p = 0.0000) | [0.08, 0.08] |
| 50-59 | 16-45 | base + race | 0.09 (p = 0.0000) | [0.09, 0.10] |
| 50-59 | 16-45 | base + BMI | 0.07 (p = 0.0000) | [0.07, 0.08] |
| 50-59 | 16-45 | base + smoke | 0.09 (p = 0.0000) | [0.09, 0.09] |
| 50-59 | 16-45 | base + BMI + smoke | 0.08 (p = 0.0000) | [0.08, 0.08] |
| 50-59 | 16-45 | base + BMI + smoke + race | 0.09 (p = 0.0000) | [0.08, 0.09] |
| 50-59 | 46-75 | base | 0.02 (p = 0.0000) | [0.01, 0.02] |
| 50-59 | 46-75 | base + race | 0.03 (p = 0.0000) | [0.02, 0.03] |
| 50-59 | 46-75 | base + BMI | 0.01 (p = 0.0000) | [0.01, 0.01] |
| 50-59 | 46-75 | base + smoke | 0.03 (p = 0.0000) | [0.02, 0.03] |
| 50-59 | 46-75 | base + BMI + smoke | 0.02 (p = 0.0000) | [0.01, 0.02] |
| 50-59 | 46-75 | base + BMI + smoke + race | 0.02 (p = 0.0000) | [0.02, 0.03] |
| 50-59 | 76-100 | base | 0.02 (p = 0.0000) | [0.01, 0.02] |
| 50-59 | 76-100 | base + race | 0.02 (p = 0.0000) | [0.02, 0.03] |
| 50-59 | 76-100 | base + BMI | 0.01 (p = 0.0001) | [0.00, 0.01] |
| 50-59 | 76-100 | base + smoke | 0.03 (p = 0.0000) | [0.02, 0.03] |
| 50-59 | 76-100 | base + BMI + smoke | 0.02 (p = 0.0000) | [0.02, 0.02] |
| 50-59 | 76-100 | base + BMI + smoke + race | 0.02 (p = 0.0000) | [0.02, 0.03] |
| 60-74 | 0-15 | base | 0.06 (p = 0.0000) | [0.06, 0.07] |
| 60-74 | 0-15 | base + race | 0.07 (p = 0.0000) | [0.06, 0.07] |
| 60-74 | 0-15 | base + BMI | 0.06 (p = 0.0000) | [0.05, 0.06] |
| 60-74 | 0-15 | base + smoke | 0.06 (p = 0.0000) | [0.06, 0.07] |
| 60-74 | 0-15 | base + BMI + smoke | 0.06 (p = 0.0000) | [0.05, 0.06] |
| 60-74 | 0-15 | base + BMI + smoke + race | 0.06 (p = 0.0000) | [0.05, 0.06] |
| 60-74 | 16-45 | base | 0.04 (p = 0.0000) | [0.04, 0.05] |
| 60-74 | 16-45 | base + race | 0.05 (p = 0.0000) | [0.04, 0.05] |
| 60-74 | 16-45 | base + BMI | 0.04 (p = 0.0000) | [0.03, 0.04] |
| 60-74 | 16-45 | base + smoke | 0.05 (p = 0.0000) | [0.04, 0.05] |
| 60-74 | 16-45 | base + BMI + smoke | 0.04 (p = 0.0000) | [0.03, 0.04] |
| 60-74 | 16-45 | base + BMI + smoke + race | 0.04 (p = 0.0000) | [0.04, 0.05] |
| 60-74 | 46-75 | base | 0.03 (p = 0.0000) | [0.02, 0.03] |
| 60-74 | 46-75 | base + race | 0.03 (p = 0.0000) | [0.03, 0.03] |
| 60-74 | 46-75 | base + BMI | 0.02 (p = 0.0000) | [0.01, 0.02] |
| 60-74 | 46-75 | base + smoke | 0.04 (p = 0.0000) | [0.03, 0.04] |
| 60-74 | 46-75 | base + BMI + smoke | 0.03 (p = 0.0000) | [0.02, 0.03] |
| 60-74 | 46-75 | base + BMI + smoke + race | 0.03 (p = 0.0000) | [0.03, 0.03] |
| 60-74 | 76-100 | base | 0.01 (p = 0.0000) | [0.01, 0.02] |
| 60-74 | 76-100 | base + race | 0.02 (p = 0.0000) | [0.01, 0.02] |
| 60-74 | 76-100 | base + BMI | 0.01 (p = 0.0030) | [0.00, 0.01] |
| 60-74 | 76-100 | base + smoke | 0.03 (p = 0.0000) | [0.02, 0.03] |
| 60-74 | 76-100 | base + BMI + smoke | 0.02 (p = 0.0000) | [0.01, 0.02] |
| 60-74 | 76-100 | base + BMI + smoke + race | 0.02 (p = 0.0000) | [0.02, 0.02] |

**Table S9a** Comparison of slopes through 2019 (sensitivity analysis) and 2022 (primary analysis): base + BMI + smoke + race and ethnicity model, Physical Well-being, BRFSS 2003 – 2022, ages 40-74

| **income** | **age** | **slope 2022** | **CI 2022** | **slope 2019** | **CI 2019** |
| --- | --- | --- | --- | --- | --- |
| 0-15 | 40-49 | 0.00 | [-0.01, 0.00] | 0.05 | [0.04, 0.06] |
| 16-45 | 40-49 | 0.03 | [0.03, 0.04] | 0.07 | [0.06, 0.08] |
| 46-75 | 40-49 | 0.00 | [-0.01, 0.00] | 0.00 | [-0.01, 0.00] |
| 76-100 | 40-49 | 0.00 | [-0.01, 0.01] | 0.01 | [0.01, 0.02] |
| 0-15 | 50-59 | 0.01 | [0.00, 0.02] | 0.05 | [0.04, 0.06] |
| 16-45 | 50-59 | 0.07 | [0.07, 0.08] | 0.13 | [0.13, 0.14] |
| 46-75 | 50-59 | 0.01 | [0.01, 0.02] | 0.02 | [0.02, 0.03] |
| 76-100 | 50-59 | 0.02 | [0.01, 0.02] | 0.04 | [0.03, 0.04] |
| 0-15 | 60-74 | 0.01 | [0.00, 0.01] | 0.03 | [0.03, 0.04] |
| 16-45 | 60-74 | 0.00 | [0.00, 0.01] | 0.02 | [0.02, 0.03] |
| 46-75 | 60-74 | 0.00 | [-0.01, 0.00] | 0.01 | [0.01, 0.02] |
| 76-100 | 60-74 | 0.01 | [0.00, 0.01] | 0.02 | [0.01, 0.03] |

**Table S9b** Comparison of slopes through 2019 (sensitivity analysis) and 2022 (primary analysis): base + BMI + smoke + race and ethnicity model, Mental Well-being, BRFSS 2003 – 2022, ages 40-74

| **income** | **age** | **slope 2022** | **CI 2022** | **slope 2019** | **CI 2019** |
| --- | --- | --- | --- | --- | --- |
| 0-15 | 40-49 | 0.07 | [0.06, 0.07] | 0.08 | [0.07, 0.09] |
| 16-45 | 40-49 | 0.09 | [0.08, 0.09] | 0.08 | [0.07, 0.08] |
| 46-75 | 40-49 | 0.06 | [0.05, 0.06] | 0.03 | [0.02, 0.03] |
| 76-100 | 40-49 | 0.06 | [0.06, 0.07] | 0.03 | [0.03, 0.04] |
| 0-15 | 50-59 | 0.04 | [0.04, 0.05] | 0.05 | [0.04, 0.06] |
| 16-45 | 50-59 | 0.08 | [0.08, 0.09] | 0.10 | [0.09, 0.10] |
| 46-75 | 50-59 | 0.04 | [0.04, 0.05] | 0.03 | [0.03, 0.04] |
| 76-100 | 50-59 | 0.04 | [0.04, 0.05] | 0.02 | [0.02, 0.03] |
| 0-15 | 60-74 | 0.07 | [0.07, 0.08] | 0.08 | [0.07, 0.09] |
| 16-45 | 60-74 | 0.06 | [0.06, 0.07] | 0.06 | [0.05, 0.06] |
| 46-75 | 60-74 | 0.06 | [0.05, 0.06] | 0.05 | [0.05, 0.06] |
| 76-100 | 60-74 | 0.04 | [0.04, 0.04] | 0.03 | [0.02, 0.03] |

**Table S9c** Comparison of slopes through 2019 (sensitivity analysis) and 2022 (primary analysis): base + BMI + smoke + race and ethnicity model, Functional Well-being, BRFSS 2003 – 2022, ages 40-74

| **income** | **age** | **slope 2022** | **CI 2022** | **slope 2019** | **CI 2019** |
| --- | --- | --- | --- | --- | --- |
| 0-15 | 40-49 | 0.04 | [0.04, 0.05] | 0.08 | [0.07, 0.08] |
| 16-45 | 40-49 | 0.06 | [0.06, 0.06] | 0.08 | [0.07, 0.08] |
| 46-75 | 40-49 | 0.02 | [0.01, 0.02] | 0.01 | [0.00, 0.01] |
| 76-100 | 40-49 | 0.02 | [0.01, 0.02] | 0.02 | [0.01, 0.02] |
| 0-15 | 50-59 | 0.07 | [0.06, 0.07] | 0.09 | [0.09, 0.10] |
| 16-45 | 50-59 | 0.09 | [0.08, 0.09] | 0.13 | [0.12, 0.13] |
| 46-75 | 50-59 | 0.02 | [0.02, 0.03] | 0.03 | [0.02, 0.03] |
| 76-100 | 50-59 | 0.02 | [0.02, 0.03] | 0.03 | [0.02, 0.03] |
| 0-15 | 60-74 | 0.06 | [0.05, 0.06] | 0.08 | [0.08, 0.09] |
| 16-45 | 60-74 | 0.04 | [0.04, 0.05] | 0.05 | [0.04, 0.05] |
| 46-75 | 60-74 | 0.03 | [0.03, 0.03] | 0.03 | [0.03, 0.04] |
| 76-100 | 60-74 | 0.02 | [0.02, 0.02] | 0.02 | [0.02, 0.03] |

**Figure S1.** Changes in physical well-being by income, age and race and ethnicity, BRFSS, 2003 – 2022


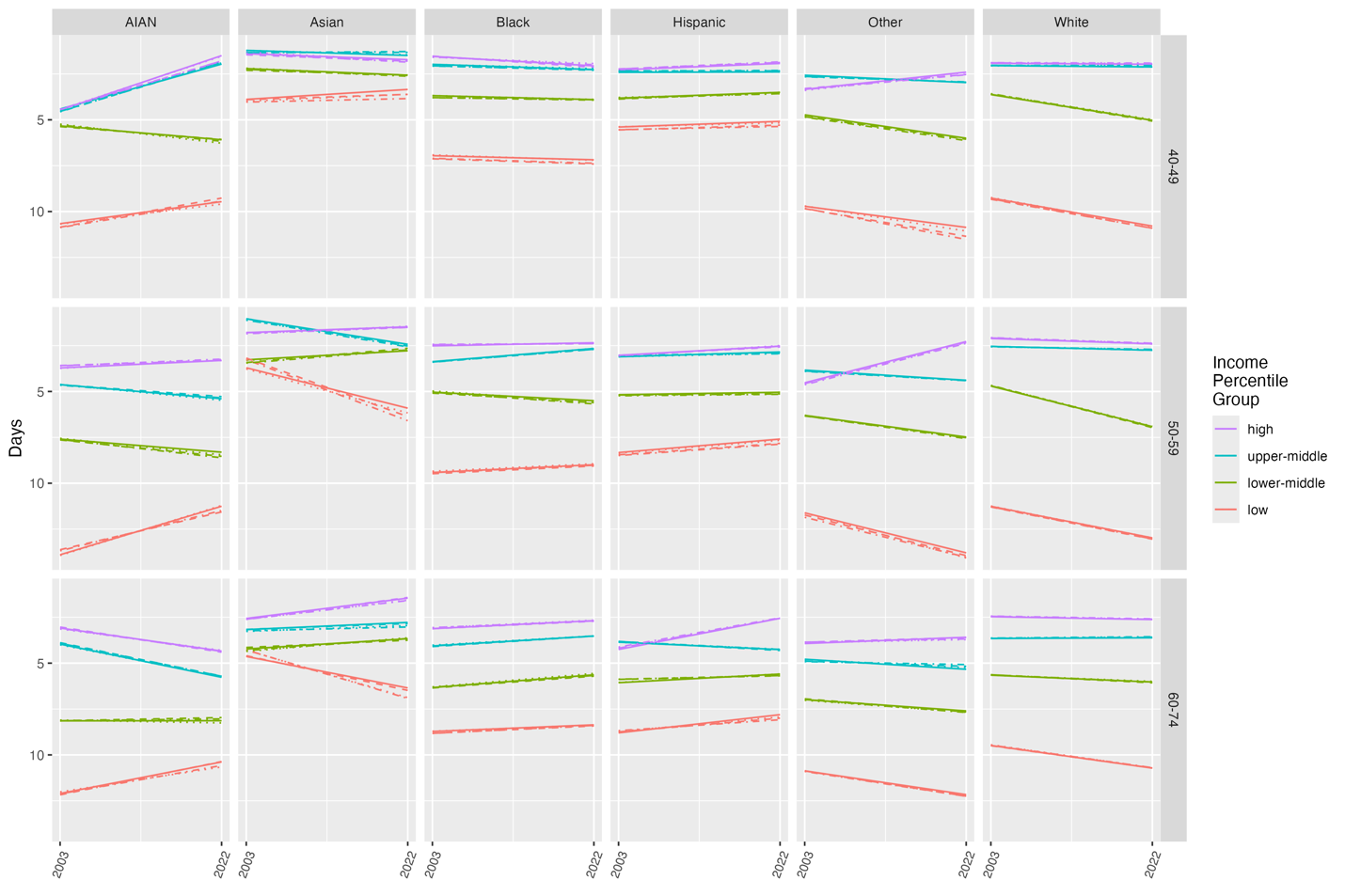


**Figure S2.** Changes in mental well-being by income, age and race and ethnicity, BRFSS, 2003 – 2022
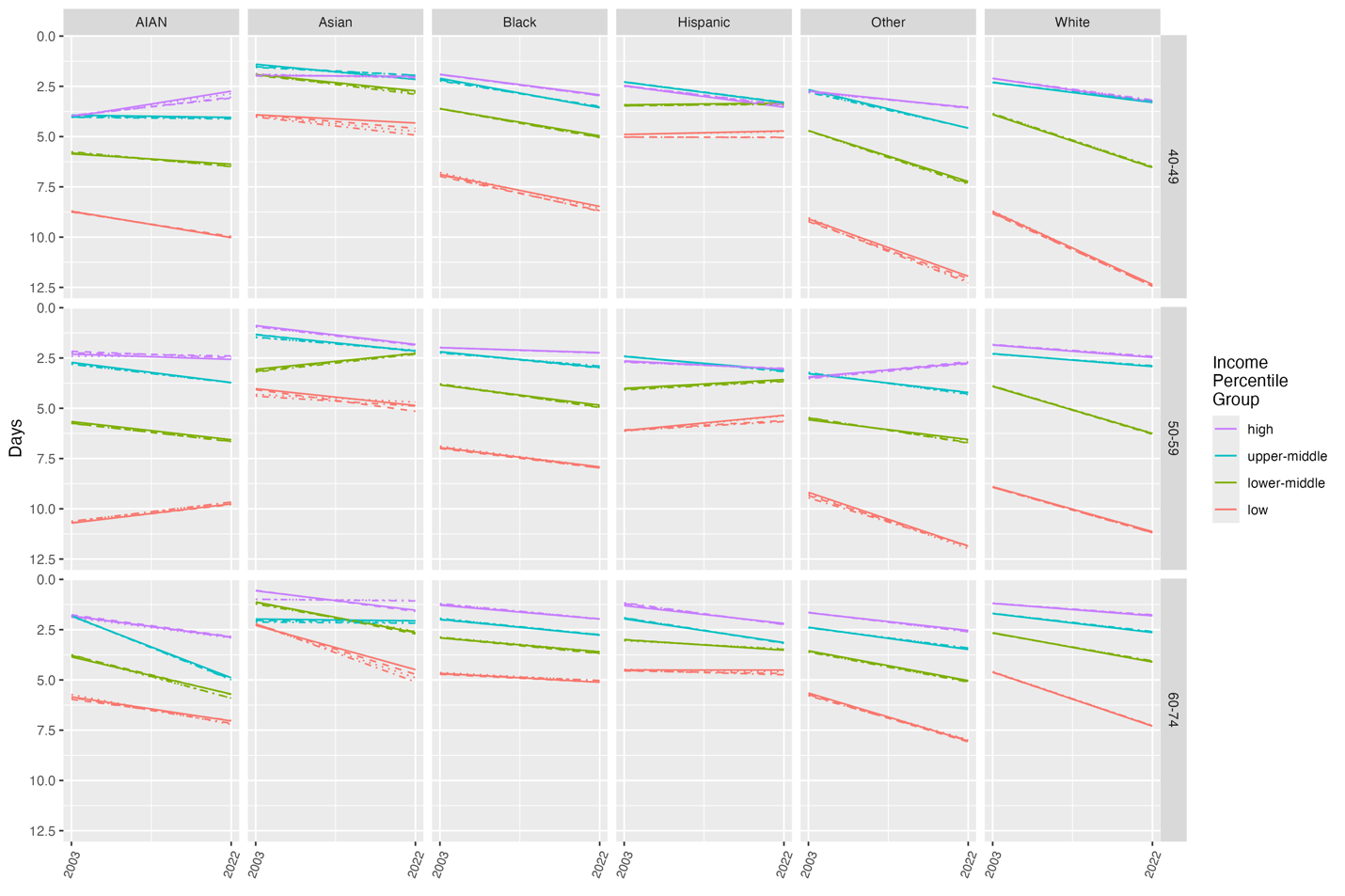


**Figure S3.** Changes in functional well-being by income, age and race and ethnicity, BRFSS, 2003 – 2022


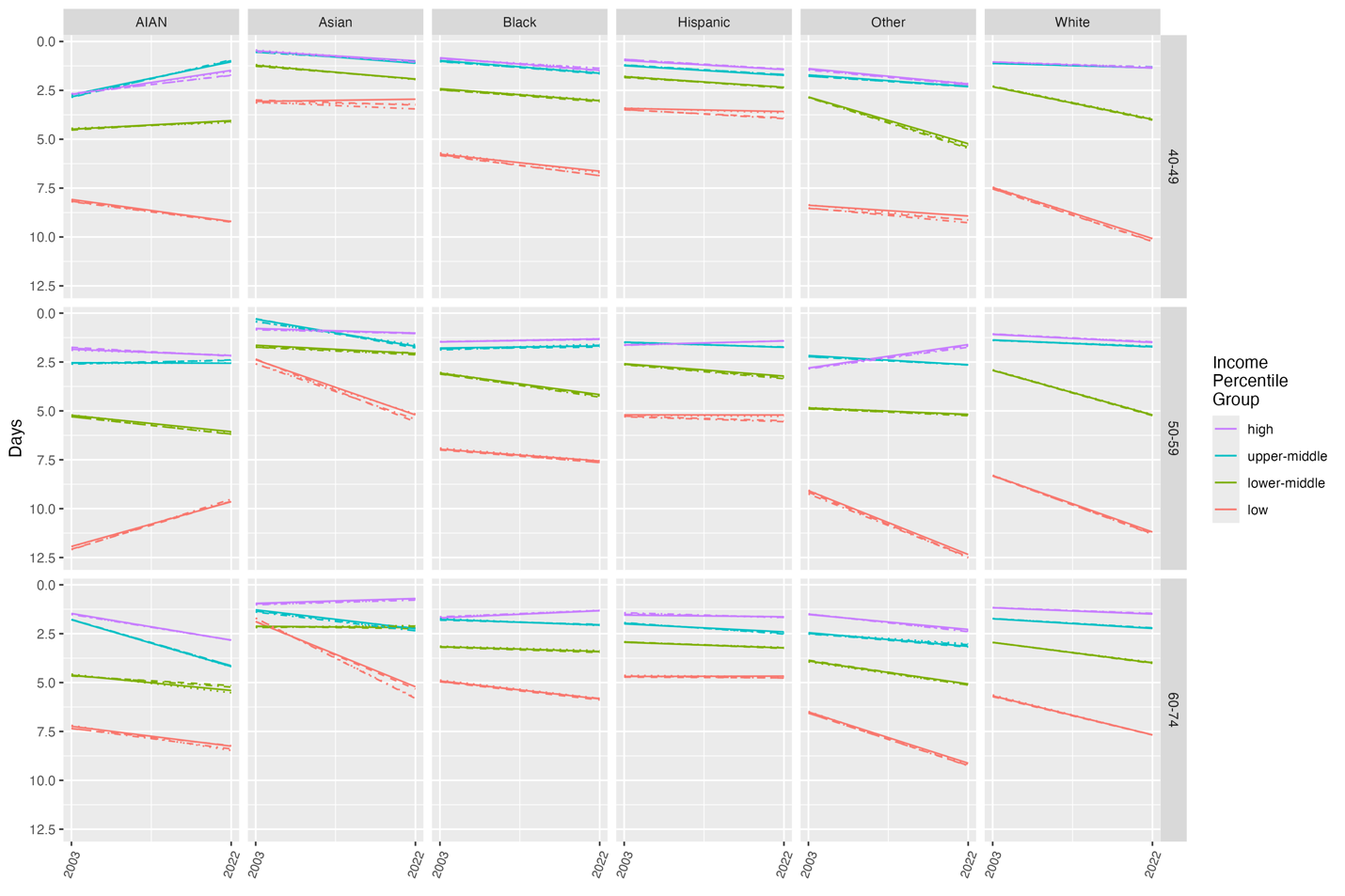

Supplement: qxae183_Supplementary_Data [file qxae183_supplementary_data.zip › HealthAffairsScholar_WellbeingTrends_Supplemental_R1.docx]
